# A Treatise on Neuralgic Diseases, Dependent upon Irritation of the Spinal Marrow and Ganglia of the Sympathetic Nerve

**Published:** 1831

**Authors:** 


Art. VII.—*A Treatise on Neuralgic Diseases, dependent upon Irritation of the Spinal Marrow and Ganglia of the Sympathetic Nerve.* By Thomas Pridgin Teale, Member of the Royal College of Surgeons in London, of the Royal Medical Society of Edinburgh, Senior Surgeon to the Leeds Public Dispensary &c.—Philadelphia: E. L. Carey & A. Hart.—pp. 115.

The term neuralgia, once limited to express a certain painful affection of the nerves, has of late been applied to designate a genus, comprehending a great variety of morbid affections of the nerves, including those which are characterized by a perverted state of the functions, as well as by severe pain.

The diagnosis and treatment of these affections have been proverbially embarrassing. This difficulty, the author thinks, has, in no small degree, originated from a mistaken view of their pathology; they having been too frequently regarded as diseases of the nervous filaments, instead of being considered symptomatic of a morbid condition of the larger nervous masses from which these filaments are derived; and the treatment, in consequence, been directed rather to the symptom than the cause.

In the more severe forms of nervous disease, paralysis, for instance, we at once recognize the brain or spinal marrow, as the seat of morbid action; but in the slighter forms of disease of the brain or spinal marrow, we overlook their probable origin, and direct our attention to the perverted or impaired action of the nervous branches.

The same pathological principle, Dr. T. thinks, may be applied to the sympathetic system of nerves, and, although it may be difficult to establish this opinion by experiment, yet we are justified, by a well-grounded analogy, in regarding the ganglionic system, as bearing the same relation to the nerves which arise from it, as the cerebro-spinal system bears to the system of nerves arising from it; and hence, many nervous affections of the viscera are to be considered, not as diseases of these viscera, but as symptomatic of disease in those particular ganglia, from which their nerves are derived.

The following is the author's description of the symptoms of *Irritation of the Spinal Marrow.*

"The symptoms of this affection consist in an infinite variety of morbid function of the nerves of sensation and volition, which have their origin in the spinal marrow, and the parts in which these morbid functions are exhibited, of course, bear reference to the distribution of the spinal nerves.

The morbid states of sensation include every variety, from the slightest deviation from the healthy sensibility of any part, to the most painful neuralgic affections on the one hand, and to complete numbness or loss of feeling on the other; including pains which may be fixed or fugitive, or darting in the direction of the nerves, prickling and tingling sensations, a sense of creeping in the skin, of cold water trickling over it, and numerous other states of perverted sensation, of which words are inadequate to convey a description. In the muscular system we find weakness or loss of power, tremors, spasms or cramps, and sometimes a tendency to rigidity.

These symptoms sometimes exist in so slight a degree, that the patient considers them unworthy of notice, and only admits their existence when particular inquiry is made respecting them; the only complaint which he makes, being of an unaccountable sense of weakness and inability of exertion. In other cases the tremors have excited alarm; sometimes the neuralgic pain in the scalp, or the fixed pain in the muscles, particularly when it occurs in the intercostal muscles, have suggested the idea of serious disease in the brain or in the lungs; and when the pain is seated in the muscles of the abdomen, a fear that some organic disease of the abdominal viscera has taken place, harasses he mind of the patient. The muscular weakness, in some cases, tending to paralysis, often suggests the fear of apoplexy, or paralysis from cerebral disease.

The affection is often of very protracted duration, undergoing alternate variations from the sanative powers of the constitution, and the different exciting causes of disease. There are many individuals in whom the complaint has existed, in varying degrees of intensity, for a series of years, without its real nature having been suspected; the patients and their medical attendants having regarded it throughout as a rheumatic or a nervous affection. * * *

In this complaint, tenderness in the portion of the vertebral column, which corresponds to the origin of the affected nerves, is generally in a striking and unequivocal manner evinced by pressure. In some instances the tenderness is so great that even slight pressure can scarcely be borne, and will often cause pain to strike from the spine to the seat of spasm or neuralgia.

This affection of the spinal marrow occasionally exists throughout its whole extent; more frequently, however, it is confined to some particular portion, and occasionally is seated in different and remote portions at the same time; the particular symptoms, and the tenderness on pressure indicating the affected part.

The symptoms, of course, vary considerably, according to the particular part of the spine which is affected, and bear reference to the distribution of the different spinal nerves.

When the upper cervical portion of the spinal marrow is diseased, we frequently find neuralgic affections of the scalp; the pain strikes in various directions over the posterior and lateral parts of the head; sometimes the twigs in the neighborhood of the ear, sometimes those which ascend over the occiput to the superior part of the scalp, are more particularly the seat of the complaint; the nervous twigs distributed to the integuments of the neck, are occasionally affected, the pain darting across the neck to the edge of the lower jaw, and sometimes encroaching a little upon the face. These neuralgic diseases frequently assume an intermittent form, the paroxysms generally occurring in the evening. A stiff neck, or impaired action of the muscles moving the head, frequently attend the affection of the upper cervical portion of the spinal marrow; and occasionally the voice is completely lost or suffers alteration, and the act of speaking is attended with pain or difficulty.

Irritation of the lower cervicle portion of the spinal marrow gives rise to a morbid state of the nerves of the upper extremities, shoulders, and integuments at the upper part of the thorax. Pains are felt in various parts of the arm, shoulder, and breast; sometimes the pain takes the course of the anterior thoracic branches of the brachial plexus, occasionally the pain is fixed at some point near the clavicle scapula or shoulder-joint, at the insertion of the deltoid, or near the elbow, or shoots along the course of some of the cutaneous nerves.—Frequently one or both of the mammæ become exquisitely sensible and painful on pressure, and some degree of swelling occasionally takes place in the breast, attended with a knotty and irregular feel, when the neuralgic pains have existed a considerable time in that part.—Prickling and numbness, tingling and creeping sensations are often felt in the upper extremities; and also a sensation of cold water trickling over the surface. On rubbing the hand over the part affected, a soreness is frequently felt, which is described as not merely situated in the integuments, but also in the more deep-seated parts. In the muscular system are observed most frequently a weakness of the upper extremities, sometimes referred particularly to the wrists, tremors and unsteadiness of the hands; also cramps and spasms of various degrees of intensity. Occasionally there is an inability to perform complete extension of the elbows, the arm appearing restrained by the tendon of the biceps; pain and tightness being produced in this part, when extension is attempted beyond a certain point. As far as I have observed, the pains and other morbid feelings in the upper extremities and chest, are felt more frequently and more severely on the left than on the right side.

Females of sedentary habits appear particularly subject to these affections of the upper extremities, and it is not uncommon for them to complain of being scarcely able to feel the needle when it is held in their fingers, and that their needle or their work frequently drops from their hands.

When the upper dorsal portion is affected, in addition to various morbid sensations similar to those in the extremities, there is often a fixed pain in some part of the intercostal muscles, to which the name pleurodynia has been assigned; and when this pain has existed a long time, there is tenderness on pressing the part. When the lower dorsal half of the spinal marrow is the seat of this irritation, or subacute inflammation, the pleurodynia when it exists, is felt in the lower intercostal muscles; frequently there is also a sensation of a cord tied round the waist; an oppressive sense of tightness across the epigastrium and lower sternal region; and soreness along the cartilages of the lower ribs, or in the course of insertion of the diaphragm. Various pains, fixed and fugitive, are also felt in the parietes of the abdomen, throughout any part of the abdominal and lumbar muscles; the pain is frequently fixed in some portion of the rectus muscle, and not unfrequently in the oblique muscle or transversalis, a little above the crest of the ilium, particularly when the origin of two or three of the lowest dorsal nerves is diseased.

The affection of the lumbar and sacral portion of the spinal cord often produces a sensation of soreness in the scrotum and neighboring integuments, and the lower extremities become the seat of various morbid sensations, spasms, tremors, &c., for the most part resembling those which have been described as occurring in the upper limbs.—The patients also complain of a sense of insecurity or instability in walking; their knees totter, and feel scarcely able to support the weight of the body.

In some cases very considerable relief is found from recumbency, the pain frequently being diminished as soon as the patient retires to bed, independently of any paroxysmal remission."

This irritation, or subacute inflammation of the spinal marrow, is not necessarily connected with deformity of the spine, or disease of the vertebræ. They may, indeed, exist together, and inflammation and ulceration of the vertebræ, or intervertebral cartilages, may predispose to, and, in some instances, act as the exciting cause of an inflammatory state of the nervous trunk, which they contain; as the two affections frequently exist together. But, although they frequently exist at the same time, they bear no regular relation to each other. Curvature of the spine seems to have no connection with the disease under consideration, since they are so seldom found together, that the former cannot even be considered as an exciting cause of the latter; and, when they do occasionally co-exist, local treatment will relieve the nervous symptoms, while the curvature remains unaltered.

The treatment, recommended by Dr. T., is very simple.

*Treatment*—When the different neuralgic symptoms which have been enumerated, can be traced to this morbid state of some portion of the spinal marrow, the treatment that ought to be pursued, is readily decided upon. Local depletion by leeches or cupping, and counter irritation by blisters to the affected portion of the spine, are the principal remedies. A great number of cases will frequently yield to the single application of any of these means. Some cases, which have even existed several months, I have seen perfectly relieved by the single application of a blister to the spine, although the local pains have been ineffectually treated by a variety of remedies, for a great length of time. A repetition of the local depletion and blistering is, however, often necessary after the lapse of a few days, and sometimes is required at intervals for a considerable length of time. In a few very obstinate cases, issues of setons have been thought necessary; and where the disease has been very unyielding, a mild mercurial course has appeared beneficial. * * *

When my attention was first directed to this subject, I considered recumbency a necessary part of the treatment; it is, for a moderate length of time, undoubtedly beneficial, and frequently very much accelerates recovery; but subsequent observation has convinced me that it is by no means essential. I have seen several instances of the most severe forms of these complaints, occurring in the poorer classes of society, where continued recumbency was impracticable, which have, nevertheless, yielded without difficulty to the other means of the treatment, whilst the individuals were pursuing their laborious avocations."

The constitutional treatment varies with the complications of the disease, but very little more will be necessary than proper attention to the condition of the digestive organs. Where there exists a disposition to relapse, Dr. T. recommends a stimulating liniment to the spine, for some weeks after the other treatment is discontinued—generally using a liniment of one part of spirit of turpentine, and two of olive oil.

The author has given a number of cases, in illustration of his principles and mode of practice. The three first were cases of intermittent neuralgia of the scalp. In these, tenderness—in the first, second, third, or fourth, cervical vertebra was detected by pressure. They were all, except the last, relieved by applying a few leeches to the tender vertebræ. In the third case, after the leeches, a blister was applied, with the effect of relieving the symptoms very speedily. Dr. T. remarks, and the remark, which we consider of some consequence, we are able to confirm by our own observation, that the pains in the head, which occur in fevers, will often be found to be of this neuralgic character, and altogether independent of any cerebral affection. A few leeches, applied to the nape of the neck, will generally produce immediate relief of this distressing symptom.

*Case* 4. Neuralgia of the upper extremities and thoracic parietes; fixed and constant pain in the neck, and between the shoulders, and darting pains from the cervical portion of the spine, over the occiput, and downward, across the neck, and over each shoulder; both arms affected with aching pains, and a sense of soreness on pressure; pricking sensations, numbness, and cramps in the forearms and fingers; occasional pain in the abdominal muscles, relieved by recumbency; no cough or difficulty of breathing. These complaints have been of long standing, and a great variety of remedies have been used for them, without benefit.

Pain having been found to exist in the two lower cervical and six upper dorsal vertebræ, leeches were directed, followed by a blister, and a recumbent position enjoined. In two weeks, the distressing symptoms were relieved, and the patient possessed a degree of muscular power, which she had not enjoyed for years. Slight occasional attacks since that period, have been relieved by the same means.

*Case* 5 we shall give in the author's words.

"Mrs. ——, æt. 48, but without having experienced any change in the catamenia, of a healthy appearance, and mother of a large family, had suffered about seven years from a painful affection of the left breast. On examination it was found to be exquisitely sensitive to the slightest touch; it was somewhat increased in size, and irregularly indurated, having a knotty feel, and an obscure sense of tumors, as if the glandular structure were enlarged at different parts. The integuments and cellular substance between the breast and clavicle, and towards the axilla, were thickened. There was a constant sense of uneasiness in the part, but her chief sufferings arose from its highly sensible state, which constantly exposed her to pain, from the irritation of her dress, or any accidental contact. Her spirits were depressed, and an apprehension that the disease would prove cancerous, although she was repeatedly assured of the contrary, was a source of great anxiety. Leeches, evaporating lotions, and warm fomentations had been employed, and medical treatment had been particularly directed to the digestive organs; these means were occasionally productive of slight alleviation, but never of permanent benefit. The complaint varied in degree, being sometimes less severe for a few weeks, without any obvious cause for the temporary amendment.

Whilst in this state, (September, 1827,) she became subject to pains in the scalp, and vertigo, attended with flatulence. The symptoms directed my attention to the spine, which on examination was found to be tender in several parts. The most painful vertebræ were the second cervical, the seventh cervical, and two upper dorsal. Leeches were applied to these parts, with considerable relief to the pains in the scalp and vertigo. Since that time she has been occasionally in the habit of applying leeches, a blister, or a sinapism, of her own accord, when there has been any return of uneasiness in the head.

On making inquiry (August 10th, 1829,) respecting the complaint in the breast, of which I had not heard any mention for several months, she tells me that from the time of her commencing the treatment by local applications to the spine, the affection of the breast has disappeared. The pain and swelling are removed, and the breast resembles the other in every respect.

The circumstance of finding a portion of the spine tender, and the removal of the tenderness by suitable remedies, being unexpectedly accompanied with relief of the fullness and pain in the breast, could not fail to produce a powerful impression on my mind, and to excite a suspicion that this irritable affection of the breast was a neuralgia of that part dependent upon disease of the spinal marrow."

After detailing another very similar case, the author remarks that the description given by Astley Cooper, of the irritable breast, exactly coincides with the phenomena attendant on these neuralgic affections.

"When," says he, "the complaint affects the glandular structure of the breast, there is scarcely any perceptible swelling, but one or more of the lobes becomes exquisitely tender to the touch; and, if it be handled, the pain sometimes continues for several hours. The uneasy sensation is not confined to the breast alone, but it extends to the shoulder and axilla, to the inner side of the elbow, and to the fingers; it also affects that side of the body to the hip," &c. "Patients also state, that heat and cold frequently succeed each other in the breast, and it would seem that the pain resembles that in tic-douloureux, darting like electricity through the part, and through the neighboring nerves."

*Cases* 7 *and* 8 were neuralgiæ of the intercostal nerves of the abdominal parietes and lower extremities—the tenderness, in the one case, existing in the second and third dorsal, and in the other, in the second and third lumbar vertebræ; relieved, as the rest, by leeches, blisters, recumbency, and a very mild constitutional treatment.

*Case 9.—Neuralgia of the Knee.*

"August 10th, 1829. E. B., æt. 18 years, a girl of a delicate constitution, and in a very humble station of life, is admitted a patient of the dispensary, on account of an affection of the left knee.

She complains of violent pain in the knee, principally situated about the ligament of the patella and in the popliteal space; from these parts the pain darts upwards along the outer side of the thigh towards the loins. It comes on suddenly for a few minutes and then leaves her free for a short time; towards afternoon the intervals of ease become shorter, until at last the pain is almost constant; during the evening it gradually diminishes, and before she retires to bed, in a great measure ceases. Whilst the pain is severe, there is considerable tumefaction of the knee, but no redness, and the skin is exquisitely sensible to the touch; in the absence of the paroxysms the swelling subsides, and the skin bears pressure without inconvenience. The pain is often accompanied with a feeling of numbness across the middle of the thigh. No affection of the right leg, or of the upper extremities. She has suffered severely during the last month, and since November, has had several attacks of a similar kind, lasting for a few weeks. The complaint was produced, she supposes, by working in a damp kitchen.

She appears in a delicate state of health in other respects, having a cough and some expectoration. The catamenia are regular. The appetite is deficient, and the bowels are relaxed and rather irritable.

She feels a great degree of aching and weariness in the lower part of the back, and the lower lumbar vertebræ are very tender on pressure."

Dr. T. would, by no means, insist that this treatment will be invariably successful, for medical science has not yet arrived at that degree of certainty, that the most correct diagnosis and the most judicious treatment will invariably succeed.

The next portion of the work is devoted to irritation of the Ganglia of the Sympathetic Nerve, from which we make the following extract:

"The ganglia of the sympathetic nerve appear subject to a state of disease similar to that which has been described in the preceding chapter, as occurring in the spinal marrow.

As the disease may be confined to one part of the spinal marrow, or may exist simultaneously in different portions, or may even pervade its whole extent, so the affection of the ganglia may be confined to one of these nervous masses, may exist in several which are contiguous, or in ganglia remote from each other; and, as there is reason to believe, the whole chain may occasionally be affected.

The disease of the ganglia is seldom found, except in conjunction with that of the corresponding portion of the spinal marrow, whereas the spinal marrow is often affected without the neighboring ganglia being under the influence of disease. Thus we frequently find symptoms of disease in a portion of the spinal marrow, without any evidence of its existence in the corresponding ganglia, frequently the symptoms of both combined, and occasionally, but rarely, symptoms referable to the ganglia, without the spinal marrow being affected.

The principal symptoms resulting from irritation of the ganglia of the sympathetic, are to be found in those organs which derive their nerves from this source. They consist of perverted functions of these organs, and are exemplified by a variety of phenomena. The *involuntary muscles*, deriving their power from the sympathetic, have their action altered, as is evinced by spasms and irregularity in their contractions. The heart is seized with palpitations, the large vessels with inordinate pulsations; the muscular fibres connected with the bronchial apparatus are thrown into spasms, constituting a genuine asthma independent of bronchial inflammation. The muscular fibres of the stomach and intestines become the seat of spasm, and of various other deviations from their natural operations. The *sensibility* of the organs, which derive their sentient power from the great sympathetic, is variously perverted, the nervous filaments being the seat of pains. The heart and lungs, for instance, are subject to morbid sensations, bearing great analogy to those which have been designated "tic douloureux," when occurring in the spinal nerves. The stomach and intestines are liable to similar neuralgia, to which the names gastrodynia and enterodynia have been applied. The kidneys, the bladder, and the uterus, are liable to the same perverted state of their sensibility. The *secretions* also undergo alteration, products being formed, which in health have no existence. This is exemplified by the enormous secretions of air which sometimes occur in the stomach. Large quantities of clear transparent liquid are also secreted by this organ, constituting what is called pyrosis. The secretions of the stomach undergo variation in their qualities, rendering them unfit for the digestive process. It is probable that the secretion of the liver also experiences some alterations in these complaints. The urine is sometimes influenced, and I am inclined to suspect that some forms of diabetes partake of a neuralgic character. Leucorrhœa is frequently a concomitant of these diseases, and ceases on their removal; but I am not prepared to say that it is ever symptomatic of them. Irregularities in the catamenia are often observed, the discharge being generally in excess.

The ganglia most liable to the disease, are the middle and lower thoracic, from which the splanchnic nerves are derived, giving rise to various disorders of the stomach and digestive organs, which will hereafter be more fully discussed. Next in frequency is the affection of the cervical ganglia, producing painful and spasmodic states of the heart. The symptoms denoting disease of other ganglia, although occasionally met with, are less frequent in their occurrence. Irritability of temper and depression of spirits often attend these complaints, particularly when the stomach is the part which suffers.

The disease of the ganglia, like that of the spinal marrow, is not necessarily connected with disease of the vertebræ or distortion of the spine. It may coexist with these complaints, and when it does so, the symptoms proper to the ganglionic disease are often erroneously supposed to be produced by distortion, or by disease of the vertebræ; they are, however, frequently relieved by treatment, whilst the disease of the bones remains uninfluenced by it, and the most extreme distortion of the spine, or destruction of vertebræ from inflammation, may exist without there being any symptoms attributable to neuralgia of the sympathetic nerves.

In conjunction with the symptoms denoting disease of the ganglia, tenderness to a greater or less degree may generally be found on pressing some part of the spine, and the tender portion invariably corresponds with the symptoms; or rather, the seat of tenderness is near the part occupied by the particular ganglia from which the nerves of the disordered organ are derived; for example, when the heart is affected, the tenderness is found in some of the cervical vertebræ, and when the stomach is the seat of complaint, it is in some of the middle or dorsal vertebræ.

With respect to the treatment, I have but little to add to what has been said in the preceding chapter respecting the treatment of irritation of the spinal marrow. Leeches, cupping, blisters, &c., to the neighborhood of the affected ganglia, constitute the essential part.—Some internal remedies are useful as adjuvants or paliatives; as quinine, iron, and other tonics, when the violence of the complaint has been subdued, and an intermittent state still continues; digitalis and prussic acid, in palpitations and unnatural pulsations; prussic acid, bismuth, and opium in gastrodynia, pyrosis, and unnatural sensibility of the stomach; and opium in severe spasms or pains in the nerves. These remedies, however, are seldom necessary; and when alone relied upon, without any treatment being directed to the affected ganglia, often fail, even of giving temporary relief. Of still less efficacy, either temporary or permanent, are external remedies, as leeches or blisters, when applied to the region of the heart or stomach, if these organs are the seat of neuralgia. I have seen cases of nervous affections of the heart, (palpitations unattended with organic disease,) which for months had resisted the employment of digitalis, blisters to the chest, and various other remedies, yield immediately to the application of a blister over the cervical portion of the spine. Numerous instances of stomach complaints, which for a length of time had been an annoyance to myself as well as others, when treated by local remedies to the epigastrium, and by medicines called stomachic, have disappeared as if by magic, on the employment of leeches or a blister over the lower dorsal vertebræ."

To illustrate these affections, of the sympathetic nerves, the author treats separately of neuralgia of the stomach, and of the heart.

Neuralgia of the heart generally comes on with palpitations, at first, occurring after exercise, or mental emotion, and soon subsiding. These paroxysms, as the disease advances, increase in frequency and violence, being excited on the slightest mental emotion, or change of position, until, at length, the irregular action of the heart scarcely ever subsides.

These palpitations are often attended by other symptoms, dependent upon the same ganglionic disease; dull, aching pains in the heart, lungs, occasionally in the aorta, or in the course of the carotid and subclarian arteries. Asthma is an occasional, though not a frequent attendant.

This affection of the ganglia is frequently attended with irritation of the spinal marrow, and hence the affections which have been just described, as symptomatic of that disease, are often present, and form a prominent feature in the description given, by the patient, of his disease. These nervous palpitations may be distinguished from those which arise from organic disease of the heart, by the absence of those symptoms which accompany a change of structure in that organ. In hypertrophy, the pulsations are more vehement and more uniform—in dilatation, they are felt over a greater extent of the chest—when there is obstruction to the circulation, from disease of the valves or other causes, the symptoms of imperfect oxygenation of the blood are present.

The nervous affection of the heart may exist in conjunction with organic disease, and under these circumstances, the symptoms will be very much palliated by remedies directed to the ganglionic system. Some have supposed that the nervous affections predispose to the organic diseases of this organ. Laennec states, however, that he has never met with any thing to countenance such a supposition.

*Neuralgia of the Stomach.* Various diseases are included under the term dyspepsia—Chronic gastritis, under which may be included all the varieties of chronic irritation, and inflammation of this organ.—Simple indigestion, apparently from mere atony of the stomach, occurring during the convalescence from other diseases.

There are, however, numerous other cases of stomach complaints very much resembling these two diseases, but resisting most obstinately the remedies generally successful in removing or palliating them. Many of these cases, which Dr. T. had unsuccessfully treated for some time, he has found to be connected with tenderness of the spinal column, and has relieved by remedies adapted to that condition. From further observation, he was led to attribute these symptoms to irritation of the ganglia, from which the splanchnic nerves are derived, and the tenderness in the neighborhood of these ganglia confirms this supposition.

The principal symptoms denoting this condition, in addition to tenderness in the neighborhood of the middle and lower thoracic ganglia, are

1st. Indigestion, and the symptoms consequent upon it.

2d. Pain in the stomach, which, when the disease is of long standing, is increased by pressure; a sense of faintness, or what is usually denominated a sinking sensation at the stomach.

3. Flatulence, not arising, in the estimation of the author, from the decomposition of undigested aliment, but from a copious secretion of air, sometimes taking place instantaneously.—Pressure on the painful vetebræ causes, in some instances, a sudden extrication of the gas.

4. Pyrosis, sometimes accompanied with gastrodynia.

5. Pulsation of the epigastrium.

6. Affections of the spinal nerves, producing a corded sensation around the waist, constriction across the epigastrium, soreness along the edges of the ribs, sometimes increased on pressure, pains in the intercostal muscles, or the muscles of the abdomen.

"On reviewing these symptoms, it will be seen that some of them are common to gastritis as well as to the affection of the ganglia, and others are more particularly characteristic of the latter. Indigestion and its consequences, as acidity, flatulence from decomposition, &c., may arise equally from disease affecting the surface, where digestion is performed, or from disease of the part where the nerves engaged in digestion originate. Pain in the epigastrium is common to both diseases, but in the nervous affection it is generally described as a dull, aching sensation; in gastritis the pain is more frequently accompanied with a feeling of heat, and is more particularly, though not always, increased by the ingestion of warm liquids. Tenderness on pressing the epigastrium more commonly attends chronic gastritis than the disease of the ganglia. The sensations of faintness and sinking seem common to both complaints. Flatulence from secretion, I believe, is seldom dependent upon gastritis, and is the most frequent of any symptom of the ganglionic affection. Pyrosis, I believe, is always attributable to the affection of the ganglia, and I think is never produced by inflammation of the mucous membrane of the stomach. The various affections of the spinal nerves, though not always attendant upon disease of the ganglia, are seldom absent, but they are never essential to, and but rarely accompany chronic gastritis; writers on dyspepsia, however, often confound these affections, and explain the spinal neuralgic symptoms on the vague principles of sympathy. In addition to these diagnostic marks, one peculiar to gastritis may be mentioned; this is the red, and sometimes prominent state of the papillæ of the tongue, particularly at its tip and edges; the tongue is also loaded, and a slight febrile state is more frequent in chronic inflammation of the stomach, than in the nervous affection.

From a careful consideration of the symptoms, it will not in most cases be difficult to establish a diagnosis, particularly when the suspicion of disease of the ganglia is corroborated by the circumstance of their being tenderness in the neighborhood of the splanchnic ganglia.

The affections which have been described, principally refer to the heart and stomach; other organs, however, I believe are occasionally affected from a similar cause; the lungs are the seat of painful and spasmodic affections, producing true asthma; the small and large intestines, the kidneys, the bladder, and uterus, are not unfrequently the seat of neuralgia, connected with tenderness in the spine, and remediable by means directed to the tender part."

Before proceeding to illustrate his principles by a report of cases, Dr. T. enters into a train of physiological and pathological observation, by which he arrives at the following conclusion; viz: That the muscular action of the heart, arteries, stomach, and intestines, depend upon the sympathetic system of nerves. 2. That the painful affections of the lungs, heart, stomach, and intestines, depend upon the same system of nerves. Lastly, that this same system of nerves, although it does not control, exerts a very consiberable influence over digestion. As the facts upon which these conclusions are formed, are already before the profession, it is not necessary for us to enter into the discussion of them.

Our limits do not permit us to enter further into the treatment of these affections, than simply to state, that it coincides with that already directed for irritation of the spinal marrow.

A considerable portion of this work is devoted to angina pectoris, a disease which the author attributes to the same source; viz, irritation or inflammation of the ganglia. With an extract representing his views upon this subject, we shall conclude our quotations from this work.

"I am fully convinced that it is to the nervous system we must look for the seat of this disease; but the great error, which has been committed by those who have assigned to angina pectoris a seat in the nerves, consists in their having overlooked the pathological fact, to which I formerly alluded, namely, that when any of the nervous masses, as the brain, spinal marrow, or ganglia, are the seat of disease, the morbid phenomena are not so much exhibited in the masses themselves as in the parts to which the nerves arising from them are distributed. Thus Laennec, whose views are by far the most definite of any that have been quoted, expressly states that he considers the seat of the disease to be in the pneumo-gastric nerve, or in the *filaments* of the great sympathetic, "dans les *filets* que le cœur recoit du grand sympatique." The treatment has also been conducted with reference to such pathological views; blisters, issues, and other remedies have been applied by the earlier writers to the neighborhood of the heart or stomach; most frequently, however, without much benefit; and Laennec has recommended magnetism, for which purpose he employed two strongly magnetized plates of steel, of a line in thickness, of an oval form, and slightly bent to the form of the chest; one of the plates he applied to the left præcordial region, the other to the opposite part of the back, so that the magnetic current might traverse *the affected part;* and when the magnet gave but little relief, he applied a blister under the *anterior* plate. Hence it is evident that his pathological and therapeutic views extended only to the nervous filaments; or, what amounts to the same thing, to the parts which were the immediate seat of pain, oppression, or morbid sensibility. On the contrary I think it would be much more consistent with actual facts, if we were to consider these local affections of the nerves merely as symptomatic of disease in the nervous masses from which they are derived; and to refer the morbid affections of the spinal nerves to disease in those portions of the spinal marrow whence they originate, and the morbid train of symptoms dependent upon the ganglionic nerves to the ganglia from which they are derived.

Upon this principle the constriction or oppression at the lower part of the thorax or epigastrium, the tightness around the waist, the pains in the lower intercostal and in the abdominal muscles, should be referred to disease of the lower dorsal portion of the spinal marrow.

The pain in the stomach, flatulence, pyrosis, &c., to disease of the lower thoracic ganglia of the sympathetic.

The pains, numbness, weakness, and the morbid sensations in the neck, breast, and upper extremities, to an affection of the cervical portion of the spinal marrow.

And the palpitations of the heart, and painful affections of the heart and lungs, to the cervical ganglia of the sympathetic.

On referring to the various cases of the angina pectoris which have been recorded, we find that the disease, in its simplest form, consists in the affection of the lower dorsal portion of the spinal marrow, attended with constriction and tightness across the epigastrium; to this is most frequently added an affection of the corresponding thoracic ganglia of the sympathetic from which the great splanchnic nerves are derived, producing distention, flatulence, and pain in the stomach; frequently there also exists an affection of the cervical portion of the spinal marrow, causing the pains, and other morbid sensations, in the neck, breast, and upper extremities, particularly on the left side, and perhaps occasionally dyspnœa from impaired action of the diaphragm; and not unfrequently there exists a similar affection of the cervical ganglia of the sympathetic, producing, in addition to the former symptoms, palpitations and painful affections of the heart and lungs.

This inflammatory affection of certain portions of the ganglionic system, and of the spinal marrow, is not unfrequently accompanied by various organic diseases of the heart itself, as ossification of the coronary arteries, of the valves, or of the aorta; softening, hypertrophy, or dilatation; producing, in addition to the symptoms peculiar to disease of the ganglia and spinal marrow, additional symptoms, peculiar to disease of the heart itself, as the more severe forms of palpitations, intermittent pulsations, obstructed circulation, and consequent lividity, anasarca, &c. From this combination we may easily comprehend why these affections so often prove suddenly fatal, and why organic diseases of the heart are so generally, though not universally, found on the dissection of those subjects who have died of angina, since the disease seldom proves fatal, unless accompanied with some organic disease of the heart. It is also possible, and even probable, that the disordered state of the nerves of the heart, from an affection of the cervical ganglia, may predispose to the organic changes in that organ which have been so frequently observed.

The paroxysmal exacerbations of the complaint depend upon undue accumulations of blood in the heart, which render it incapable of acting with sufficient energy to propel the blood with freedom, particularly when its muscular structure is in a state of atony, or atrophy, from ossification of the coronary arteries, fatty degenerescence or softening; or when there exists any obstruction at the different apertures. And it is not improbable that, when the nerves of the heart are simply affected from disease of the ganglia and a consequent state of nervous palpitation without organic disease of the heart exists, that the muscular structure may be so weak and irritable as to be unable to act with that regularity and decision which is necessary for maintaining a regular and equable transmission of the blood. The causes which excite these paroxysms are such as accelerate the circulation or determine the blood more rapidly to the heart than it has the power of transmitting; as passions of the mind, exercise, and that state of the system which of late has been termed congestion, in which the blood ceases to circulate freely in the superficial parts of the body and in the extremities, and is accumulated in some of the internal organs; a state attended with coldness and pallor of the surface, and oppressed functions of those organs which are more particularly the seat of the congestion. From any of these causes blood may be accumulated in the heart and large vessels, and, when the muscular apparatus of this organ is impaired by any alteration of structure or by disease of its nerves, it may be unable, at least for a time, to restore an equable state of the circulation; a sense of anxiety, of pain, or of fulness in the region of the heart, and an impression that life is actually on the point of being suspended, is the result. In this state, after ineffectual efforts to propel its contents, the heart, as it were, feels its inability, and sometimes yields to a temporary paralysis or syncope, and, not unfrequently, its tone is so completely overpowered, that it remains for ever paralysed.

The earlier writers applied the term angina pectoris only to the more severe forms of the affection, from which arose the opinion that it was a disease of extreme danger. Daily observation, however, abundantly testifies that it may exist in every degree of violence, from the slightest sense of oppression and flatulence, to the inexpressible anxiety, constriction, and suffocation, which mark the more aggravated forms of the disease, particularly when they are combined with an organic affection of the heart. On this subject I am glad to find my views correspond with those of Laennec, who considers that in a slight degree it is a disease of very frequent occurrence.

I have been induced to refer the various groups of symptoms which have been described as angina pectoris, to an affection of some portion or portions of the spinal marrow, and of the corresponding ganglia of the sympathetic, by the following considerations.

1. The fact, as I have before observed, that most of the morbid phenomena exhibited in the extreme filaments of nerves, are seldom owing to disease in the nerves themselves, but to an affection of the nervous mass from which they are derived.

2. The coexistence of pain on pressing some portion of the spine, with the symptoms constituting angina pectoris; and the correspondence of the painful part of the spine with the particular symptoms which are present; namely, tenderness in the lower dorsal portion of the spine in conjunction with the stomach affection, constriction, &c., and tenderness in the cervical spine, with pains in the arms, breast, and shoulders, and palpitations.

3. The relief obtained by local antiphlogistic measures to the spine; for instance, to the lower dorsal portion when the stomach is affected; and there is constriction, and to the cervical portion when there is an affection of the arms and palpitations."

It is scarcely necessary for us to recommend the author's views to the attention of the profession, since the perplexity which our brethren have doubtless all experienced in the treatment of diseases of this nature, will at least induce them to grasp at every prospect of relief, however remote. In our last number, we gave an account of Dr. Tate's observations on Hysteria, to which he gives a similar location. In every case of this disease, which has since fallen under our observation, this tenderness has been found to exist, and very generally the disease has been found to yield to remedies directed by this view of its cause.—That the same practice will apply to a very great variety of nervous diseases, we have no doubt; and if others find the same benefit from it, that we have, some of the most distressing, and hitherto, obstinate diseases, to which the human frame is liable, will be found comparatively easy in their management.

